# Medical ethics of long-duration spaceflight

**DOI:** 10.1038/s41526-023-00333-9

**Published:** 2023-11-28

**Authors:** Siddharth Rajput, Ivy Mayor, Madison Diamond, Mark Rosenberg, Victor Cole, Nikita Bhakare, Omar Aziz, Anderson L. Wilder

**Affiliations:** 1 Advanced trainee in Vascular surgery, Royal Australasian College of Surgeons, Sydney, Australia; 2 Space Generation Advisory Council (SGAC), Sydney, Australia; 3 Karolinska Institute, Stockholm, Sweden; 4 Space Generation Advisory Council (SGAC), Stockholm, Sweden; 5 Department of Space Studies, University of North Dakota, Grand Forks, ND USA; 6 Department of Neurology, Medical University of South Carolina, Charleston, SC USA; 7 Centre for Biomedical Ethics, Yong Loo Lin School of Medicine, National University of Singapore, Singapore, Singapore; 8 University of Pune, Pune, India; 9 Royal Australasian College of Surgeons, Sydney, Australia; 10 Department of Psychology, Florida Institute of Technology, Melbourne, FL USA

**Keywords:** Medical ethics, Health policy

## Abstract

With the advent of novel and emerging technologies, long duration spaceflight will become more common; along with it, an increase in its inherent health risks. However, health-related ethical issues arising during long-duration spaceflight remain poorly characterized, uncertain and unpredictable. Medical ethics is defined as a set of moral principles, beliefs and values that guides choices about medical care. This set of principles, founded in our sense of right and wrong, helps us make fair and just decisions. The paper conceptually and analytically investigates the ethical issues likely to arise from medical complications during spaceflight, mapping unfilled gaps of the current status quo. Furthermore, this paper explores broad ethical themes of autonomy, nonmaleficence, beneficence and justice, while also delving deeper into specific scenarios within each theme. The manuscript represents an up-to-date review of the available literature in the field of space medical ethics and recommends guiding ethical principles and a framework for their application to negotiate the resolution of complex ethical scenarios during long-duration spaceflight.

## Introduction

Humanity has always explored the unknown – from extended naval exploration voyages to Yuri Gagarin’s historic spaceflight in 1961. Exploration comes with risks to human life – known and unknown. Yet, this has never stymied humanity’s indomitable quest for the unknown. As we move beyond Low Earth Orbit (LEO) and deeper into uninhabitable and hostile space environments, we must revisit the balance between our quest and the ethics of such explorations.

Human spaceflight poses significant risks. Health risks are varied throughout the different mission phases, with some emerging and resolving rapidly in-flight (e.g., space motion sickness) and others causing long-term health damage post-flight (e.g., cancer due to radiation exposure). With long-duration missions, the risks are naturally amplified^1^. NASA’s Human Research Program (NASA HRP) focuses its research on investigating and mitigating the highest risks to astronaut health and performance in support of exploration missions. Each health risk is evaluated by an integrated team of risk custodians that assesses a Likelihood and Consequence (LxC) rating and risk disposition. At the time of writing, cardiovascular adaptations, food and nutrition, and spaceflight associated neuro-ocular syndrome remain ‘red risks’ (on a ‘green - yellow - red’ scale), while vestibular and sensorimotor impacts and sleep and circadian misalignment have been moved to ‘yellow risks’^2^.

Based on conceptual considerations and analysis, the following paper offers a way of contextualizing the ways in which familiar ethical principles may be applied in long-duration space exploration (LDSE). Moreover, the paper will identify and discuss the relevant moral principles that should aid the formation of an ethical framework for in-flight space medicine decision-making, in the context of known health challenges in space and scenarios where ethical standards are not defined. This process has been guided by a narrative review of the pertinent literature.

## Results

### Principles of medical ethics

Ethics are best described as the guiding principles for construction of a moral life, while morality refers to collective norms of right or wrong human conduct within a society. In medicine, professional morality specifies general moral norms for the practice of medicine. These moral norms or standards drive our values, and our values drive our behaviours. Our professionalism reflects our values and behavioural standards, thus defining our ethical standards^3^. In its simplest form, *medical ethics* is defined as a set of moral principles, values, and beliefs governing medical practice. Ethics provides structure to standardize ethical decision-making, much akin to the structure grammar provides to any language^4^.

Medical ethics is an ever-evolving, experience-based, dynamic and applied discipline dedicated to the norms and principles that should guide professional medical practice^5^. Beauchamp and Childress define four ethical principles that serve to guide professional ethics, namely^6^,

1. Respect for Autonomy or the norm of respecting the decision-making capacities of autonomous persons.
2. Nonmaleficence or the norm of doing no harm.
3. Beneficence or a group of norms for providing benefits.
4. Justice or a group of norms for distributing risks, benefits, and costs fairly.

Respect for Autonomy is a foundational principle of ethics and refers to respecting the self determination of individuals and acknowledges their right to hold views, to make choices, and to take actions based on personal values and beliefs^6^. Such respect involves respectful action, not merely a respectful attitude. In the context of spaceflight, there are two possible applications. Firstly, an astronaut providing consent during LDSE. The primary justification for advancement of informed consent is to protect autonomous choice. Second, privacy and confidentiality of health information are predicated upon respect for people’s autonomy in controlling information about themselves.

The principle of Nonmaleficence asserts an obligation not to inflict harm on others. In medical ethics, it has been closely associated with the maxim of “Primum non nocere – Above all do no harm”^6^. In the context of spaceflight, all efforts should be made, based on current knowledge, to mitigate known risks and to plan for unknown risks. Campbell et al. discussed the ethical issues around prophylactic surgery before an extended mission^7^. Specifically, the authors considered the ethics of removing healthy organs (appendix and gallbladder) for unclear potential benefits while exposing the astronaut to possible surgical complications, namely, adhesional bowel obstruction or altered gut immune function.

The principle of Beneficence in medical ethics refers to the moral obligation to act for the benefit of others. In medical ethics, we acknowledge that to prevent and remove harm, and to weigh and balance an action’s possible good against its costs and possible harm, is important in the practice of medicine.

Justice is interpreted as fair, equitable, and appropriate treatment in light of what is due or owed to persons^6^. While equality involves the equal division of a limited resource, equity involves the division of resources based on a person’s needs. Justice in the context of spaceflight, involves equal risk distribution for in-flight duties, such as extravehicular activities or activities involving a risk of radiation exposure. The individual variation in a particular risk may be known or unknown, for example, the stochastic and deterministic effects of radiation in men and women.

In addition to the core principles, moral virtues like compassion, integrity, and conscientiousness which form part of virtue ethics, support and enrich the moral framework of medical ethics. Human death and Futility are essential concepts in terrestrial medicine and are relevant in LDSE as well. The natural human instinct is to survive; hence, on an instinctual level, death is never the answer. The common societal perception that one must do everything to avoid death further drives this instinct, and this, in turn, leads to futile interventions. There needs to be a keen appreciation for the moment when medicine reaches the limits of its ability to alter the course of the disease. This limit may be further lowered in LDSE by factors such as the absence of specialized expert care, limited resources, and cognisance of mission success in the spaceflight environment. The concepts of futility and death really provide further means of reflecting on the challenges of ethical decision making in LDSE rather than providing ethical imperatives themselves. Weighing in the imperative of completing a mission in determining futility illustrates how the context of LDSE serves to bring into sharp relief the normative aspect of futility^8^.

### Current ethical standards for healthcare in space

This section describes the existing regulations and guidelines, and the current state of medical ethics, applied on the ISS, in Europe, and at NASA. While there is no specific framework or standard of care for LDSE, there are current medical standards and models that exist for LEO spaceflight^9^. For the ISS, “the standard of care […] is to support the crew 24/7 from Mission Control and to stabilize and transport an astronaut to Earth for definitive medical care”^9^. The ISS has a medical checklist in Russian and English that details standard medical procedures astronauts may need to perform^9^. Furthermore, astronaut health is continuously monitored, and expert medical advice is available on demand. The latter can guide a non-physician astronaut through treatment processes. Although ethics are not explicitly addressed, they can be considered implied in some areas. While it is essential to have these ethical parameters built into medical standards, a more comprehensive and targeted approach to medical ethics, and particularly, to in-flight medical decision-making, is found to be lacking.

NASA’s Office of the Chief Health and Medical Officer had asked the Institute of Medicine’s Aerospace Medicine and Medicine in Extreme Environments Committee to develop a framework for dealing with medical ethics on LDSE^1^. This has since been incorporated into a NASA procedural requirement (NPR 8900.1B), effective from December 2016 until December 2022, several months ago at the time of writing^10^. This further emphasizes the need to develop a universal space medicine ethics framework that is up to date with new, relevant data.

This group developed four recommendations. First, they recommended expanding current policies for revising health standards. Second, they suggested that the ethics principles (i.e., avoid harm, beneficence, favourable balance of risk and benefit, respect for autonomy, fairness, and fidelity) be used when expanding said health standards. Third, they recommended formally ensuring ethical responsibilities when dealing with health standards on LDSE via policy changes. This recommendation involves providing astronauts with the required information to make informed decisions, continually updating health standards as data are gathered, seeking expert advice from beyond NASA, communicating with stakeholders about decisions regarding health standards, implementing more equal opportunities in selection criteria, providing lifetime healthcare for astronauts, and protecting the privacy and confidentiality of astronauts’ health data. The final recommendation was to implement an ethics-based decision framework which has been divided into three levels involving assessment of whether the astronaut can ethically take on risks that exceed current health standards. If it is decided that they can, each mission should be assessed on a case-by-case basis, all while ensuring that the astronaut is appropriately informed to make their own decisions about participation. Furthermore, emphasis is placed on the crew being composed of the most qualified individuals to mitigate the risks as realistically as possible^1^.

No other international space agency has published LDSE health standards or medical ethics policies in English. Both the European Space Agency [ESA]^11^ and European Commission^12^ have investigated the critical elements required to sustain human health, well-being, and performance efficiency during deep-space missions. However, no European medical ethics guidelines for prolonged missions have been publicly formulated.

The Institute of Medicine’s three-level framework of assessment^1^ provides a reasonable approach for decision-making, with an emphasis on health standards and the acceptability of future missions based on a risk assessment of novel health hazards. The ensuing discussion fundamentally provides practical guidance in the ultimate development of a decision-making framework for in-flight medical dilemmas, considering the astronaut’s role as ethical decision-maker.

## Discussion

Long-duration human missions raise concerns about the crew’s well-being, dignity and rights. Given the associated risks of physical or mental disease, medical emergency, and death, all stakeholders should agree upon common and binding ethical standards and medical guidelines. Even though astronauts who volunteer for prolonged spaceflight are willing to accept known occupational health risks, ethical implications extend beyond mere personal choice. Presently, many of these health risks are not yet fully understood, and technologies for controlling them are still being developed. Astronaut healthcare standards and a medical ethics framework for LDSE should evolve in parallel with technological advances in preparation for Lunar or other extra-terrestrial endeavours.

There is an opportunity for all space agencies to develop a common ethical framework for use by governments, industries, and individuals. For instance, an interdisciplinary, international panel of experts could be formed and could regularly meet to discuss and update this framework as more information about the health risks of LDSE emerges. Such a framework should be underpinned by the shared goals to minimize risks, safeguard astronaut health and uphold ethical principles.

The medical ethics framework put forth for NASA^1^ focuses on the ethics of conducting missions that lay outside of currently accepted health standards, including a decision-making process based in risk management. The following discussion is focused on ethical considerations for in-flight clinical decision-making. As such, these foundational principles, upon which a broader framework could be built, complement the prior report. Should a mission be deemed ethically acceptable based on the prior report’s framework, the principles outlined below would provide the baseline ethical decision-making framework for in-flight medical decision-making.

Although terrestrial standards of care may be different, medical ethical principles in space remain largely unchanged. In the context of a limited set of medical capabilities that are constrained by mass, volume, and stability, it is still likely that the medical system for exploration will be able to adequately address the most serious health effects of a long duration mission. The main areas of difference between terrestrial and LDSE medicine are:

- the limited resources,
- the impacts on the entire crew,
- the availability of immediate, around-the-clock, real-time medical expertise,
- the overall level of medical care achievable, and
- the environmental conditions

Astronauts on LDSE missions will not have access to anything close to the range of tools available for treatment at a hospital or the supplies of a fully equipped medical suite. Therefore, the ethical framework must include guidance on resource allocation, not diagnostic but therapeutic. Ethical dilemmas in spaceflight will arise when limited resources impact decisions on whether to provide care and to what capacity.

The framing of the deep-space medical ethics standards must consider not just the life of a single astronaut but also the future safety and protection of other crew members. The consumption of non-reusable resources (e.g., antibiotics) to improve health outcomes for one astronaut may deplete this resource entirely, with serious or fatal consequences in a future emergency. Furthermore, the loss or severe impairment of one crew member can psychologically impact the rest of the crew, diminishing their ability to continue the mission safely. If an astronaut wishes to undergo a procedure that their crewmates disagree on, resources will be depleted and harmful side effects may occur, including interpersonal tension within the crew. This, in turn, will impact crew cohesion and collaborative performance. As described below the decision-making principles must be altered when considering the impact of one astronaut’s medical care on the crew.

In regard to available medical expertise, crews on deep-space missions are expected to be trained in and possess the skills needed to deal with medical emergencies in space. Therefore, it is paramount that during training, each crew member’s roles and responsibilities are made clear. All astronauts receive medical training. However, there is a need for specialized training on different types of equipment, accompanied with regular refreshers throughout the mission. Currently, each ISS expedition has assigned flight surgeons. Day-to-day issues are worked real-time by the flight surgeons and biomedical engineers at the Mission Control Center via teleconference, private audio or video conference, and/or meetings with the Medical Operations team and ISS crew. Long-duration missions will require an on-board chief medical officer to handle both routine medical check-ups and issues of emergent care that might arise while out of contact with ground resources^13^. The chain of command related to who must act and how medical issues are dealt with must be established early on, and medical simulations must be regularly incorporated into training. In the event the chief medical officer becomes incapacitated the next crew member in line must be identified and contingencies for other potential scenarios developed.

Decisions on withdrawing care for patients who require resource-intensive treatments may be expected much earlier than what is standard on Earth or even LEO. In light of the inherent health risks, medical care standards, medical ethics framework, and the likelihood of death occurring in a resource-limited environment, it is essential to understand that a deep-space mission is not comparable to similar scenarios on Earth.

In addressing the health standards, the more limited level of medical care will also differ from those for current missions to the ISS. The Exploration Medical Capability (ExMC) Element of the Human Research Program (HRP) has developed definitions and example actions through five levels of medical care with progressing capability from space motion sickness and basic life support to autonomous advanced life support and surgical care. ExMC acknowledges that the level of care should be considered for each mission, that each mission is made up of multiple phases that occur in different locations in space and durations, and that each phase should be assessed for the most appropriate level of care^14^.

The framework must contextualize the circumstances and how these standards are adopted. Given the current lack of data for long-term missions beyond LEO, ethically, the approach in which standards are addressed could only be adjusted on a mission-by-mission basis, considering these missions as grounds for building data and pushing the envelope. This is like the framework the Institute of Medicine provided to NASA, where they claimed that making exceptions to the current standards is the only feasible option under the present circumstances, due to a lack of data upon which to base the new standards^1^. In the future, as these data are gathered, the standard of care and acceptance of medical risks should be based on mission criteria, i.e., distance from Earth, mission duration (time in transit and on planetary bodies), accessibility of resources, and more. These should also differ for novel and routine missions. More than minimal risks could be justifiable as part of exploratory missions travelling to new areas of deep space, insofar as all crew members are fully informed and provided new information as it becomes available out of respect for their decision-making autonomy. Ensuring crew are comprehensively trained and up-to-date on how to use scarce medical resources also supports the fair distribution of both human skills and material resources in ways that yield the most benefit to individuals and the crew. Future missions with similar aims and end points should adopt the most ethically justifiable mission course, once established, after careful balancing of all risks, benefits and justice considerations. As such, the exploration of space can still be undertaken without undermining the most comprehensive medical ethical standards, at the time. One must then consider whether health standards will become more lenient. Because the topic of health standards has been covered in depth by the framework put forth to NASA^1^, it remains outside of the scope of this paper.

The following decision-making principles consider the context of LDSE and the unique circumstances astronauts will encounter on these missions, and are founded on the ethical pillars of autonomy, nonmaleficence, beneficence, and justice. These principles should act as guidance for building an ethical framework for space medicine. Importantly, one must consider that all decisions will ultimately lie with the crew. They must embody the role of medical ethics decision-maker, and as such, must be provided with comprehensive guidance that remains flexible to the unknown nature of human space exploration.

### Principle 1. Decision-making principles must be founded on the pillars of medical ethics

Although more significant risks can be accrued in spaceflight exploration than on current missions to the ISS, this cannot be at the expense of ethics. When dealing with in-flight medical scenarios, the core ethical pillars of decision-making must always be considered. In particular, risk-benefit analyses should be founded on these principles while evolving alongside the ever-changing context of LDSE. Medical decisions made at any stage (i.e., pre-flight, in-flight, post-flight) must consider impacts over time, from an ethical perspective. Importantly, this also encompasses long-term impacts on the entire crew, potentially impacting future missions and opportunities.

### Principle 2. The framework must include guidance for dealing with circumstances where the core ethical principles are in conflict or remain ambiguous

Part of ethics is understanding that there are almost endless variations in circumstances and interests that prevent the principles from being absolute and inviolate. When faced with conflicting principles on LDSE, the crew will have to examine the respective weights of the competing prima facie obligations of an ethical principle based on both content and context. For example, ensuring autonomy in the traditional sense is not always feasible and may conflict with the remaining pillars of beneficence, nonmaleficence, and justice. For instance, an unfortunate trauma during an extravehicular activity that renders a crew member unable to function or, even worse, necessitate major limb amputation and the subsequent rejection of medical care by the affected crew member, for any reasons namely personal values or cultural, can put the rest of the team or the whole mission at risk. Further, meeting mission objectives, landing on a planetary body and the implications for future missions can all impact decision-making. The ethical framework must implement an unbiased decision-making toolkit focused on the crew as a single entity.

### Principle 3. The chain of command must be established, maintained, and updated on a mission-by-mission or even case-by-case basis

There is a need to contextualize the chain of command in the setting of LDSE or how it may differ to the existing LEO/short duration missions. Medical decision-making would typically fall on the crew medical officer, the crew commander, and the flight surgeon on Earth. However, on LDSE, the chain of command will vary depending on the nature and phase of the mission and also on the level of medical care in question, especially when the normal chain of command is disrupted and non-medical crew members are suddenly responsible for medical/ethical decisions. The chain of command is important as the use of all resources on one incident may prevent future minor ailments from being treated, therefore jeopardizing human life, mission success, or both. A chain of command guides a clear directive for mission success, mitigating apprehensiveness and indecisiveness, providing crewmembers the ability to make split-second decisions in scenarios of high stress, potential cognitive impairment, and other unusual circumstances.

### Principle 4. Crews must be regularly trained on this framework (i.e., pre-flight and in-flight refreshers), and this training must include medical simulations with various ethical dilemmas

Several factors contribute to medical-ethical dilemmas: medical knowledge/expertise, negotiating complex decision-making, and values. Although astronauts receive medical training for health risks or conditions they are likely to encounter, they may lack the clinical expertise that medical professionals inherently gain from years of formal training and education. Although a medical professional is likely to be part of the LDSE crew, it is not guaranteed they will be available for medical decision-making (e.g., they are incapacitated). Medical training also focuses on non-technical skills, such as complex decision-making, which should go hand-in-hand with space crew medical training. Engaging in medical simulations can improve patient outcomes by ensuring that decisions are made through the lens of the guiding pillars of medical ethics^15,16^. One must also consider the impact of values - personal, cultural, or professional - and how those can impact decision-making. This is exemplified in cultural differences where some may value life above all else (prioritizing treatment over palliative care), whereas others may value quality of life (prioritizing end of life care over treatment)^17^. While this example lies outside the spaceflight domain, it demonstrates how values may inform a person’s decisions surrounding medical risks in space.

### Principle 5. The framework must address how to deal with conflicts and guide in-flight decision-making

While the four ethical principles inform decision making, their real value is to contextualize and guide conflicts with in-flight medical decision making in LDSE. These conflicts may relate to ethical principles (covered in principle 2) or decision makers (chain of command covered in Principle 3) or may be unrelated to either of these. As described previously, there are many potential medical scenarios in space where ethical decisions are not clearly defined. For example, a crew member could delay seeking treatment or disclosing an ailment in order to avoid postponing a mission milestone, such as an EVA. While their decision upholds their autonomy, it does not align with nonmaleficence and justice (should this decision have further-reaching impacts on the crew). Furthermore, the chain of command (Principle 3) may make these decisions more complex. Therefore, providing guidance regarding disagreements can aid decision-making when faced with complex, stressful and time-sensitive scenarios. It would be important for conflict resolution guidance to emphasize how the weighing and balancing of prima facie duties within a LDSE context differs from a terrestrial and even ISS context. As previously discussed, collective interests and mission priorities hold weight alongside autonomy and well being of individuals, and the crew as a unit. The framework could provide guidance on resolving conflicts in the form of case studies which are sufficiently nuanced and realistic as to provide good, usable examples of practical ethical decision making/conflict resolution. However as Beauchamp and Childress state, “Some moral conflict is inevitable and cannot always be avoided or eliminated by even tightly knit specifications”^6^.

### Principle 6. The ethical framework must address fidelity in all its forms

The IOM report addresses fidelity as it relates to health standards and the responsibility of the space agency to ensure long term care for astronauts after they return to Earth. However, fidelity may have additional implications in the context of LDSE. Fidelity addresses a person’s responsibility to be loyal and truthful in their relationships with others. It also includes promise keeping, fulfilling commitments and trustworthiness. A separate way in which fidelity may apply to actual decision making in LDSE is in relation to what astronauts owe to one another, particularly when assuming the role of medical decision makers (including situations when medical ethical decisions may have to be made by non-medical crew members). LDSE mission exigencies may require withholding information, violating confidentiality, or breaking promises. Astronauts would need to understand that a duty of fidelity in a collective and informed decision making within a crew is not absolute and may be defeasible e.g, in light of greater considerations for the wellbeing of the crew/mission success. In conclusion, there remains a lack of ethical guidelines for space medicine, particularly for LDSE missions. As these missions are slated to occur within the decade or so, an accurate, current and robust medical ethical framework must be developed. Simulations of medical ethical issues incorporating features of LDSE (i.e. communication delays with Earth and resource limitations) should be performed on the ISS. Therefore, any issues that do arise can be addressed before missions to the Moon, Mars and beyond. Medical ethical guidelines for LDSE should be developed by an advisory board of international experts, consisting of bioethicists, medical researchers, and space policy makers and representing all spacefaring nations. Such ethical guidelines or framework should be integrated and incorporated into international treaties such as the Outer Space Treaty and the Artemis Accords. The international community of space faring nations, through close collaboration, will need to ensure that LDSE missions, whether governmental or commercial, should make all efforts to uphold this framework in their planning and execution.

## Methods

### Data collection

We conducted a narrative review, performing searches on relevant databases (MEDLINE, PubMed, EMBASE, and Google Scholar). The search terms “ethics,” “bioethics,” “spaceflight,” and “space medicine” were searched by the authors using strategies developed through consulting team members and published literature on the subject of biomedical reviews^18,19^. Before being finalized, the methodology was refined and peer-reviewed using the PRISMA checklist. A copy of the final strategy used is available in the supplementary material.

### Literature review

The sources we have drawn on for this article included all available review articles, commentaries, studies, meeting summaries, conference proceedings, and technical reports submitted to national space agencies, addressing medical ethics policy, current practices, or standards for spaceflights. References cited in the selected publications were followed up and included where appropriate. No date restrictions were set. We excluded non-English language articles and articles with similar, but no additional relevant information. Through a screening and assessment process conducted independently by all authors to ensure quality of evidence, we identified a single publication^1^ that fully met the search criteria, highlighting a significant paucity of literature addressing this important topic.

### Reporting summary

Further information on research design is available in the Nature Research Reporting Summary linked to this article.

### Supplementary information

Reporting Summary

CHECKLIST ITEM

## Data Availability

All relevant data is available from the authors.
